# Clinical efficacy and mechanism exploration of Moxibustion for diminished ovarian reserve: a randomized controlled trial protocol

**DOI:** 10.3389/fpubh.2026.1805995

**Published:** 2026-07-16

**Authors:** Yumeng He, Yun Duan, Yang Song, Wei Zhou, Xia Chen, Zhongyu Zhou, Weiqing Kong, Kun Yang, Hong Zhou, Feng Hu, Chengwei Fu

**Affiliations:** 1 College of Acupuncture and Orthopedics, Hubei University of Chinese Medicine, Wuhan, China; 2 Department of Ultrasound, Hubei Provincial Hospital of Traditional Chinese Medicine, Wuhan, China; 3 Department of Ultrasound, Affiliated Hospital of Hubei University of Chinese Medicine, Wuhan, China; 4 Department of Radiology, Hubei Provincial Hospital of Traditional Chinese Medicine, Wuhan, China; 5 Department of Radiology, Affiliated Hospital of Hubei University of Chinese Medicine, Wuhan, China; 6 Acupuncture and Moxibustion Center, Hubei Provincial Hospital of Traditional Chinese Medicine, Wuhan, China; 7 Hubei Provincial Clinical Research Center for Acupuncture and Moxibustion in Obesity Treatment, Wuhan, China; 8 Acupuncture and Moxibustion Center, Affiliated Hospital of Hubei University of Chinese Medicine, Wuhan, China; 9 Hubei Shizhen Laboratory, Wuhan, China; 10 Department of Orthopedic Rehabilitation, Taihe Hospital, Shiyan, China; 11 Department of Orthopedic Rehabilitation, Affiliated Hospital of Hubei University of Medicine, Shiyan, China; 12 Department of Cardiovascular Medicine, Wuhan Hospital of Traditional Chinese and Western Medicine, Wuhan, China

**Keywords:** diminished ovarian reserve, fMRI, Moxibustion, randomized controlled trial, study protocol

## Abstract

**Background:**

Diminished ovarian reserve (DOR) is a condition that severely impacts female fertility, and there are currently no effective treatment strategies for DOR. Moxibustion may offer a non-invasive, low-risk intervention. This study aims to assess the clinical efficacy of moxibustion in improving ovarian reserve in women with DOR and to explore its underlying central neural mechanisms using resting-state functional magnetic resonance imaging.

**Methods:**

This prospective, single-center, parallel-group randomized controlled trial will be conducted at the Hubei Provincial Hospital of Traditional Chinese Medicine. Participants diagnosed with DOR will be randomly assigned in a 1:1 ratio to either an intervention group or a control group. They will both receive a lifestyle intervention. In addition, the intervention group will receive 16-week Yin-Yang Regulating Moxibustion. The primary outcome include changes in antral follicle count. Secondary outcomes include serum levels of anti-Müllerian hormone, follicle-stimulating hormone, and luteinizing hormonechanges in menopausal symptoms, anxiety, depression, and quality of life. Rs-fMRI will be used to investigate the brain’s central neural mechanisms.

**Discussion:**

The results of this study will provide new insights into the adjunctive treatment of DOR and elucidate its potential mechanisms through neuroimaging.

**Clinical trial registration:**

https://itmctr.ccebtcm.org.cn/, identifier ITMCTR2025001808

## 1 Introduction

Diminished ovarian reserve (DOR) is a condition in women of reproductive age characterized by a reduced response to ovarian stimulation (1). It is typically identified by decreased anti-Müllerian hormone (AMH), reduced antral follicle count (AFC), and elevated basal follicle-stimulating hormone (FSH) levels (2).

DOR affects approximately 10% of women, often due to genetic, environmental, or lifestyle factors, and its prevalence exceeds 50% in women over 40 (3). As a major risk factors for female infertility, DOR accounts for approximately 10% of all cases of female infertility (4). In addition to impairing reproductive potential, DOR is associated with increased psychological burden, and diminished quality of life. Without timely intervention, DOR may progress to premature ovarian insufficiency (POI) or premature ovarian failure (POF), posing a serious threat to women’s reproductive health (2, 5).

Current therapeutic strategies for DOR remain limited and lack universally accepted clinical guidelines. Assisted reproductive technology (ART) is commonly employed to address infertility associated with DOR. However, ART procedures are costly and may impose a significant financial burden on patients. Moreover, due to the reduced quantity and compromised quality of oocytes in DOR patients, ART is often associated with lower fertilization rates and diminished pregnancy outcomes. More concerningly, accumulating evidence suggests that offspring conceived via ART may be at increased risk for adverse outcomes, including small-for-gestational-age birth, low birth weight, preterm birth, and congenital heart defects (6). Pharmacological interventions (e.g., coenzyme Q10, DHEA, GH, and sex hormones) aim to improve ovarian response and embryo quality, but their effectiveness remains debated and may be restricted by side effects or contraindications, including potential cancer risks (7–9). Therefore, there is an urgent need to develop safe, effective, and low-risk non-pharmacological interventions for the clinical management of DOR.

Moxibustion, a traditional Chinese medicine (TCM) therapy, refers to the ignition of moxa at acupoints or in specific areas. Through warmth and near-infrared stimulation, the active components of moxa penetrate the skin, stimulate meridians, and exert therapeutic and preventive effects on a variety of conditions (10). Compared to general moxibustion therapies, ginger-partitioned moxibustion (GPM), which combines the thermal effects of moxibustion with the pharmacological properties of ginger, may exert enhanced efficacy. Ginger is widely used in TCM and has been reported to possess antioxidant and free radical scavenging activities. Under the thermal stimulation of burning moxa, its bioactive constituents may be absorbed transdermally, enhancing the overall therapeutic effect in combination with moxibustion (11). In the present study, we adopted Yin-Yang Regulating Moxibustion (YYRM), a form of GPM that applies a compressed moxa discover a ginger medium placed on selected acupoints, including Mingmen (GV4) and Shenque (CV8).

Clinical studies have suggested that moxibustion may help regulate menstrual cycles, restore hormonal balance, improve ovarian blood flow, and increase pregnancy rates in women with ovarian dysfunction (12). However, most of the evidence has been limited to hormonal changes, improvements in clinical symptoms. Beyond these limitations, a recent study indicated that moxibustion may enhance the antioxidative defense system and inhibit apoptosis in naturally aging ovaries (13). While this provides additional biological evidence, such findings remain focused on peripheral ovarian effects and do not elucidate the central mechanisms by which moxibustion may exert its therapeutic actions. Currently, the mechanism by which moxibustion affects ovarian function is believed to involve regulating the hypothalamic–pituitary-ovarian (HPO) axis, leading to an improved ovarian microenvironment (12, 14). However, most of these research conclusions are derived from animal models.

From a neuroendocrine perspective, ovarian function is regulated through coordinated interactions between the central nervous system and peripheral endocrine organs, commonly conceptualized within the HPO axis. Previous studies suggest that DOR is not only a consequence of peripheral ovarian impairment, but may also involve altered central neuroendocrine regulation (15, 16). Dysregulation of hypothalamus-centered neural networks may influence gonadotropin secretion patterns and ovarian responsiveness, thereby contributing to ovarian reserve decline and providing a rationale for exploring central regulatory pathways using functional magnetic resonance imaging (fMRI).

fMRI, as a neuroimaging method based on blood-oxygen-level-dependent (BOLD) signals, reflects functional connectivity between different brain regions by monitoring changes in blood oxygen concentration in active brain areas (17). With unique advantages such as no radiation, non-invasiveness, high-quality imaging, and high spatial resolution, it has become one of the most widely applied tools for exploring brain function. Resting-state fMRI (rs-fMRI), one of the main types of fMRI, refers to the spontaneous neuronal activity in brain regions captured during the subject’s wakeful but resting state without specific cognitive tasks (18). Compared to task-based fMRI, rs-fMRI provides a better understanding of the brain intrinsic and spontaneous neural activity.

Direct DOR-specific neuroimaging evidence regarding hypothalamus-centered functional connectivity remains limited. Available DOR-related neuroimaging studies have begun to use rs-fMRI to investigate central functional alterations in patients with diminished ovarian reserve, and hypothalamus-centered functional connectivity frameworks have also been proposed in acupuncture-related DOR research (15, 19). These studies provide preliminary methodological support for applying rs-fMRI in this field, although direct evidence regarding hypothalamus-centered functional connectivity abnormalities in DOR remains insufficient. Evidence from POI studies may also provide indirect background information for ovarian dysfunction research (20). However, POI and DOR are distinct clinical entities with different degrees of ovarian dysfunction, and POI-related findings should not be directly extrapolated to DOR. In the present study, the hypothalamus was selected as a predefined seed region primarily because of its central role in reproductive neuroendocrine regulation within the HPO axis. Therefore, in the present study, the hypothalamus-centered, hypothesis-driven seed-based rs-fMRI design should be interpreted as an exploratory approach to investigate potential central regulatory changes associated with YYRM and ovarian reserve, rather than as confirmation of an established DOR-specific central pathological model.

In the assessment of ovarian reserve, AMH and AFC are both established markers with complementary clinical value. Previous evidence has shown broadly comparable predictive performance between AMH and AFC in predicting ovarian response (1, 21, 22). In the present study, AFC was selected as the primary outcome because it directly reflects the recruitable follicle pool and has been adopted as a primary endpoint in previous acupuncture-related DOR trials (23). In addition, FSH and LH were included as secondary endocrine outcomes because they are commonly used in the clinical evaluation of ovarian reserve and have been reported in previous studies of acupuncture interventions for DOR (24). Together, these indicators provide complementary information on ovarian reserve and reproductive endocrine status in this study.

This study aims to evaluate the clinical efficacy and feasibility of moxibustion combined with lifestyle intervention for DOR through a randomized controlled trial. In addition, rs-fMRI will be employed to explore the potential central neural changes associated with the therapeutic effects of moxibustion in DOR.

## 2 Method

### 2.1 Study design and setting

**Figure 1:**
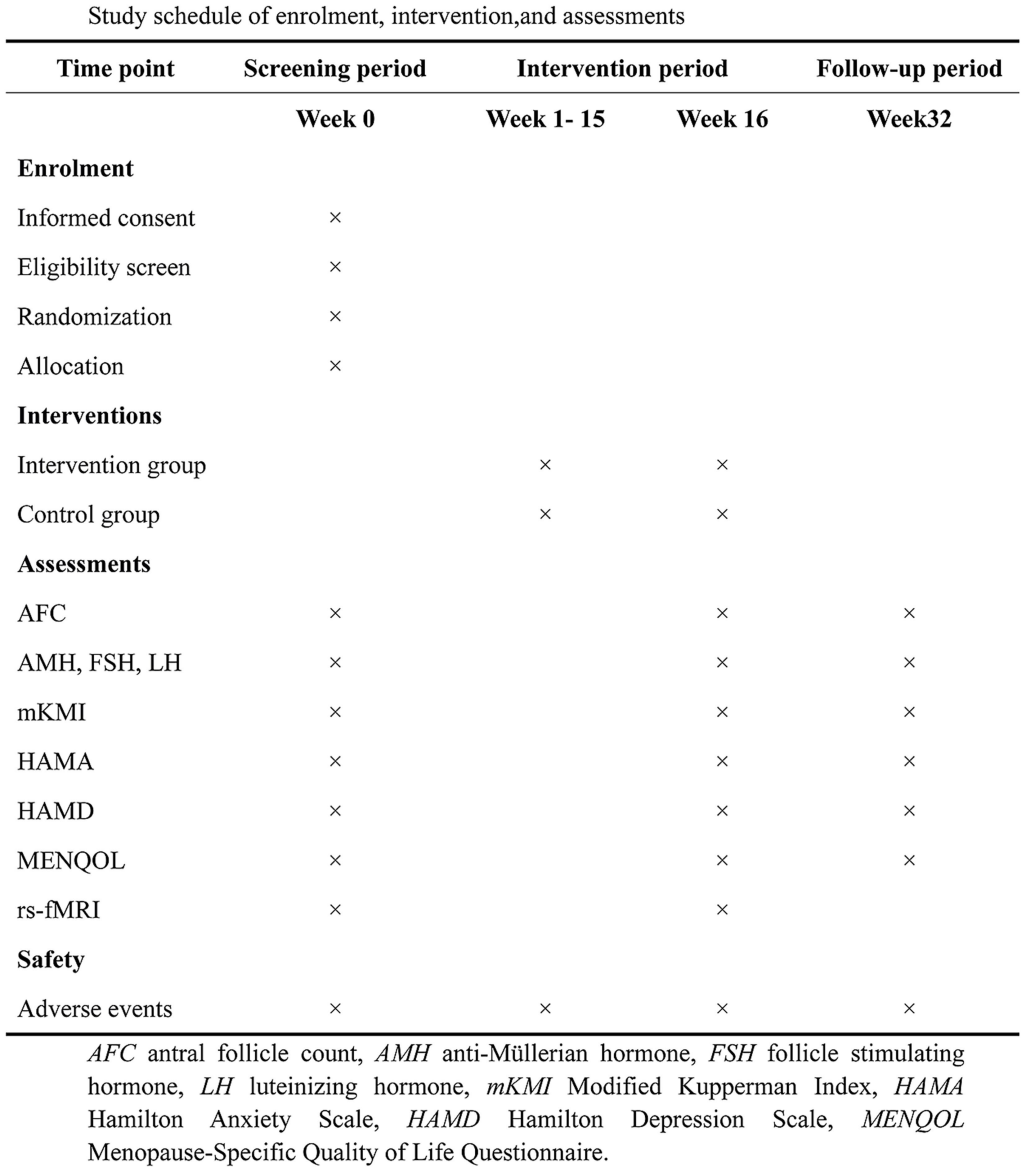
Study schedule of enrolment, interventions, and assessments.

**Figure 2:**
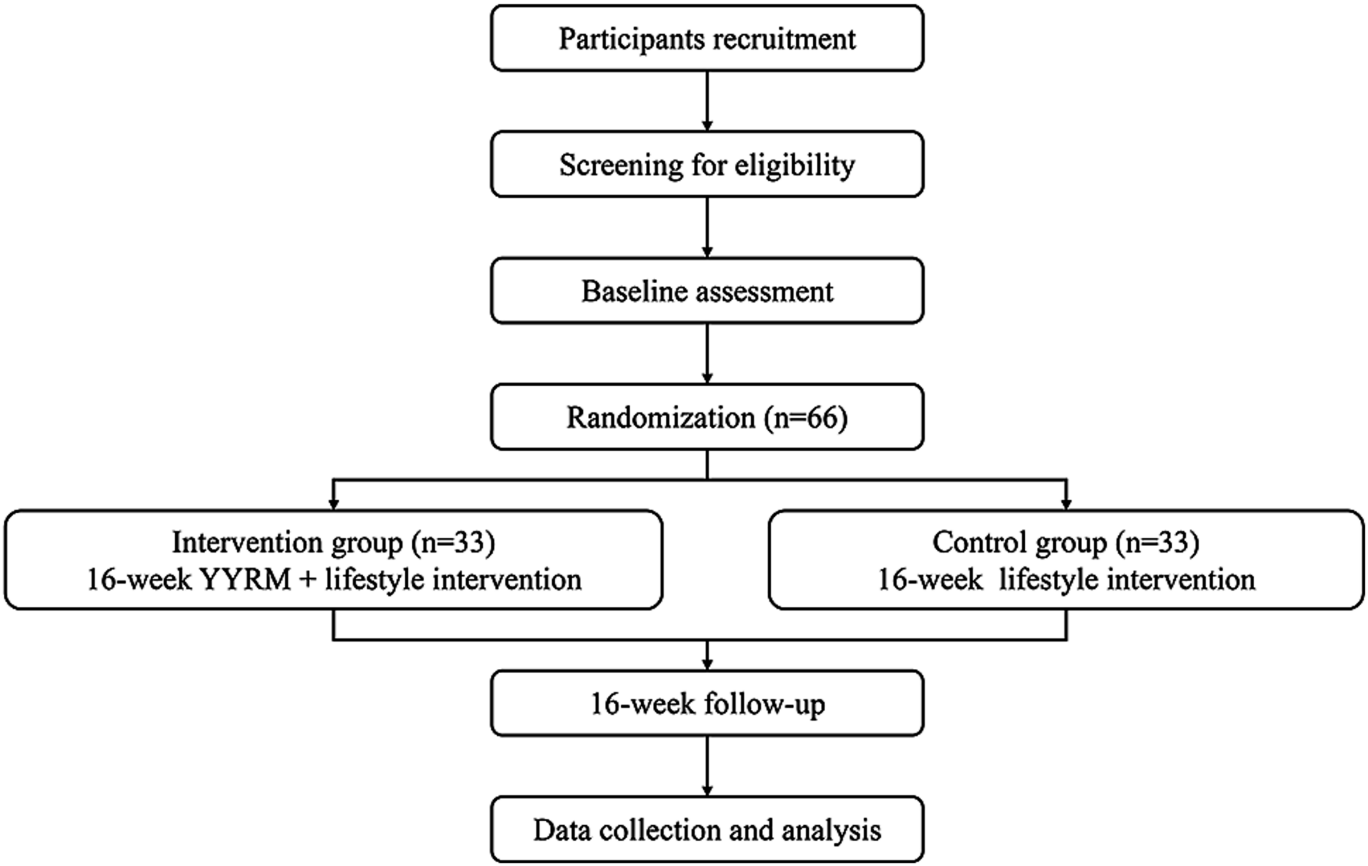
Flowchart of the study.

This prospective, single-center, parallel-group randomized controlled trial will be conducted at the Hubei Provincial Hospital of Traditional Chinese Medicine, including both outpatient and inpatient settings. The protocol follows the guidelines set forth in the Standard Protocol Items: Recommendations for Interventional Trials (SPIRIT) (25) for Interventional Trials (see Supplementary S1 File), and the results will comply with the Consolidated Standards of Reporting Trials (CONSORT) (26) standards for reporting trials. The study will span a total of 33 weeks, comprising a 1-week baseline period, a 16-week intervention phase, and a 16-week follow-up phase. The study flowchart and the schedule of assessments are presented in Figures 1, 2, respectively.

### 2.2 Ethical approval and consent to participate

The trial follows the principal ethical guidelines of the Helsinki Declaration and has received approval from the Ethics Committee of Hubei Provincial Hospital of Traditional Chinese Medicine (Ethic Approval NO. HBZY2025-C54-02). Written informed consent form will be obtained from all participants prior to enrollment. Our study has been registered in the International Traditional Medicine Clinical Trial Registry (ITMCTR2025001808, https://itmctr.ccebtcm.org.cn/). The results of the study will be published in a peer-reviewed academic journal.

### 2.3 Trial status

This clinical trial is presently undergoing participant recruitment. The first participant was enrolled on May 19, 2025, following trial registration, with the enrollment period expected to be completed by December 2027. If participant recruitment is not completed within the planned timeframe, we will consider extending the recruitment period or increasing the number of centers as appropriate.

### 2.4 Participants

#### 2.4.1 Diagnostic criteria

According to the Bologna Conference standards of the European Association for Human-Assisted Reproduction, *the Guidelines Outlined in the Expert Consensus on the Clinical Diagnosis and Management of Diminished Ovarian Reserve 2022*, the diagnostic criteria for DOR include the following:

1. Anti-Müllerian hormone (AMH) < 1.1 ng/mL;
2. Antral follicle count (AFC) < 5–7 follicles in both ovaries during the early follicular phase (days 2–4 of the menstrual cycle)
3. Basal follicle-stimulating hormone (FSH) ≥ 10 IU/L in two consecutive menstrual cycles.

#### 2.4.2 Inclusion criteria

1. Female, aged 18 to 40 years, right-handed;
2. Meets the diagnostic criteria for DOR;
3. Able to undergo transvaginal ultrasound examination;
4. Voluntarily participate in the research and sign an informed consent form.

#### 2.4.3 Exclusion criteria

Participants meeting any of the following criteria will be excluded:

1. History of polycystic ovary syndrome (PCOS), hyperprolactinemia, pituitary or hypothalamic amenorrhea, congenital reproductive tract malformations, or thyroid dysfunction as indicated by previous examinations;
2. History of ovarian surgery, such as oophorectomy or ovarian tumor resection; history of chemotherapy or pelvic radiotherapy; history of immunosuppressant use;
3. Combined with severe comorbidities affecting the cardiovascular, hematopoietic, neurological, or psychiatric systems, as well as diabetes mellitus, hypertension, etc.; hepatic dysfunction or renal dysfunction as indicated by previous examinations;
4. Use of estrogen, progesterone, or dehydroepiandrosterone within 2 months before enrollment;
5. Local skin damage or impaired thermosensation at the intervention site and any other condition that may render the participant unsuitable for acupuncture or moxibustion;
6. Receipt of acupuncture or moxibustion intervention within the past 3 months;
7. Evidence of organic brain lesions (e.g., cerebrovascular disease, epilepsy, or intracranial tumors) on MRI;
8. Cognitive impairment;
9. Participation in other clinical research trials within the past 6 months;
10. Other reasons judged by the investigator that the patient is unlikely to comply with the protocol or is unsuitable for any reason (e.g., frequent prolonged business trips, etc.).

### 2.5 Randomization, allocation and blinding

Randomization sequence will be generated using SPSS 24.0 and sealed in sequentially numbered, opaque envelopes. These envelopes will be delivered to an independent researcher responsible for outcome assessment. After obtaining written informed consent from eligible participants, the envelopes will be opened in sequence, and 66 participants diagnosed with DOR will be randomly allocated in a 1:1 ratio to either the intervention group (*n* = 33) or the control group (*n* = 33) based on the assignment enclosed to receive a 16-week intervention, respectively. Due to the particular nature of moxibustion research and practical clinical considerations, blinding of clinicians and participants is not feasible. To minimize potential biases, outcome assessments, data processing, and statistical analyses were conducted by independent third-party evaluators who were not involved in treatment implementation and were unaware of group assignments throughout the study period.

### 2.6 Intervention

Participants in both groups will receive lifestyle intervention during the 16-week intervention period. According to previous evidence from reproductive medicine (27–29), participants will be encouraged to adopt a balanced diet, engage in moderate physical activity suited to individual capacity, and maintain regular work and rest schedule. They will also be advised to avoid smoking, limit alcohol consumption. We designed a healthy lifestyle adherence scoring to assess adherence (see Supplementary S2 File). Lifestyle adherence will be assessed weekly throughout the 16-week intervention period using the Healthy Lifestyle Adherence Scoring System, which includes four domains: dietary modification, physical activity, sleep–wake regulation, and avoidance of harmful substances. Each domain will be scored from 0 to 2, with a total weekly score ranging from 0 to 8. Deviations from lifestyle recommendations will be documented during follow-up assessments.

#### 2.6.1 Intervention group

**Figure 3:**
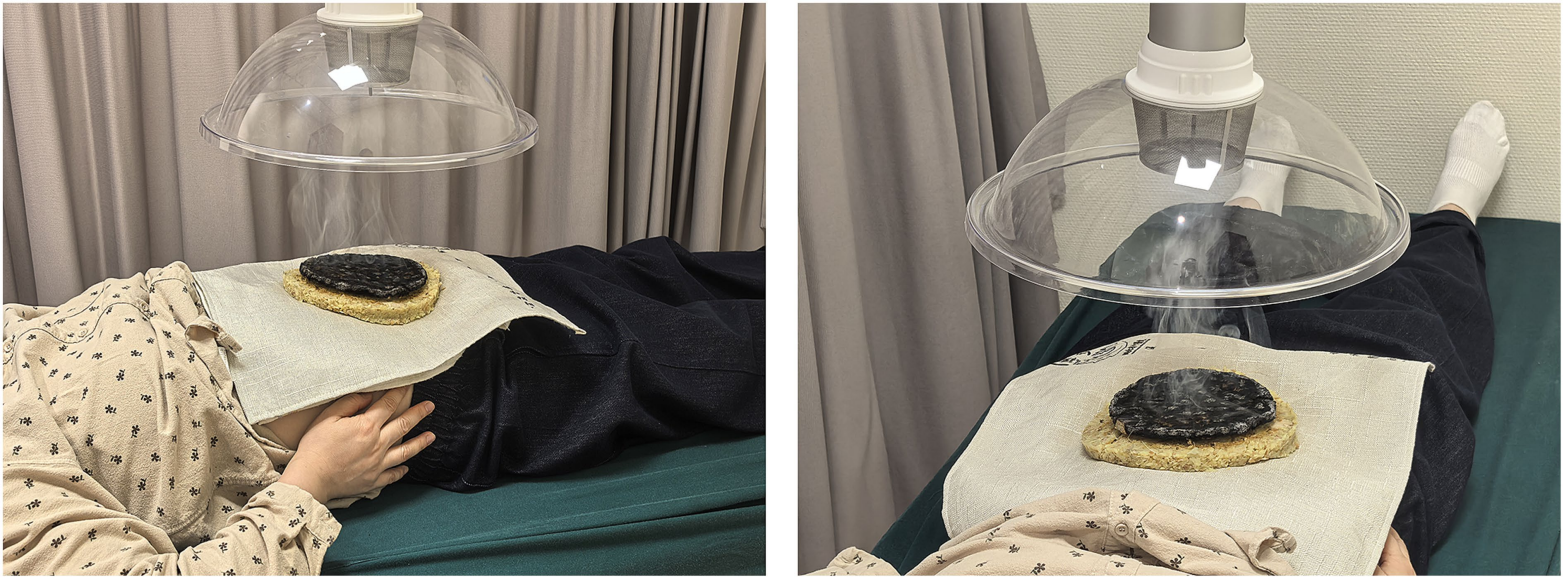
Fixed two-bed treatment room for Yin-Yang Regulating Moxibustion. The room is equipped with dedicated moxibustion smoke purification and local exhaust devices to provide standardized environmental control during treatment.

Participants in the intervention group will receive a standardized YYRM therapy once weekly for 16 weeks. All treatments will be administered in a fixed treatment room equipped with a dedicated moxibustion smoke purification and local exhaust device (Hubei Aite Environmental Purification Technology Co., Ltd., Hubei, China) (Figure 3). This therapy alternates between prone and supine positions, with moxibustion applied to specific body regions based on traditional meridian theory.

**Figure 4:**
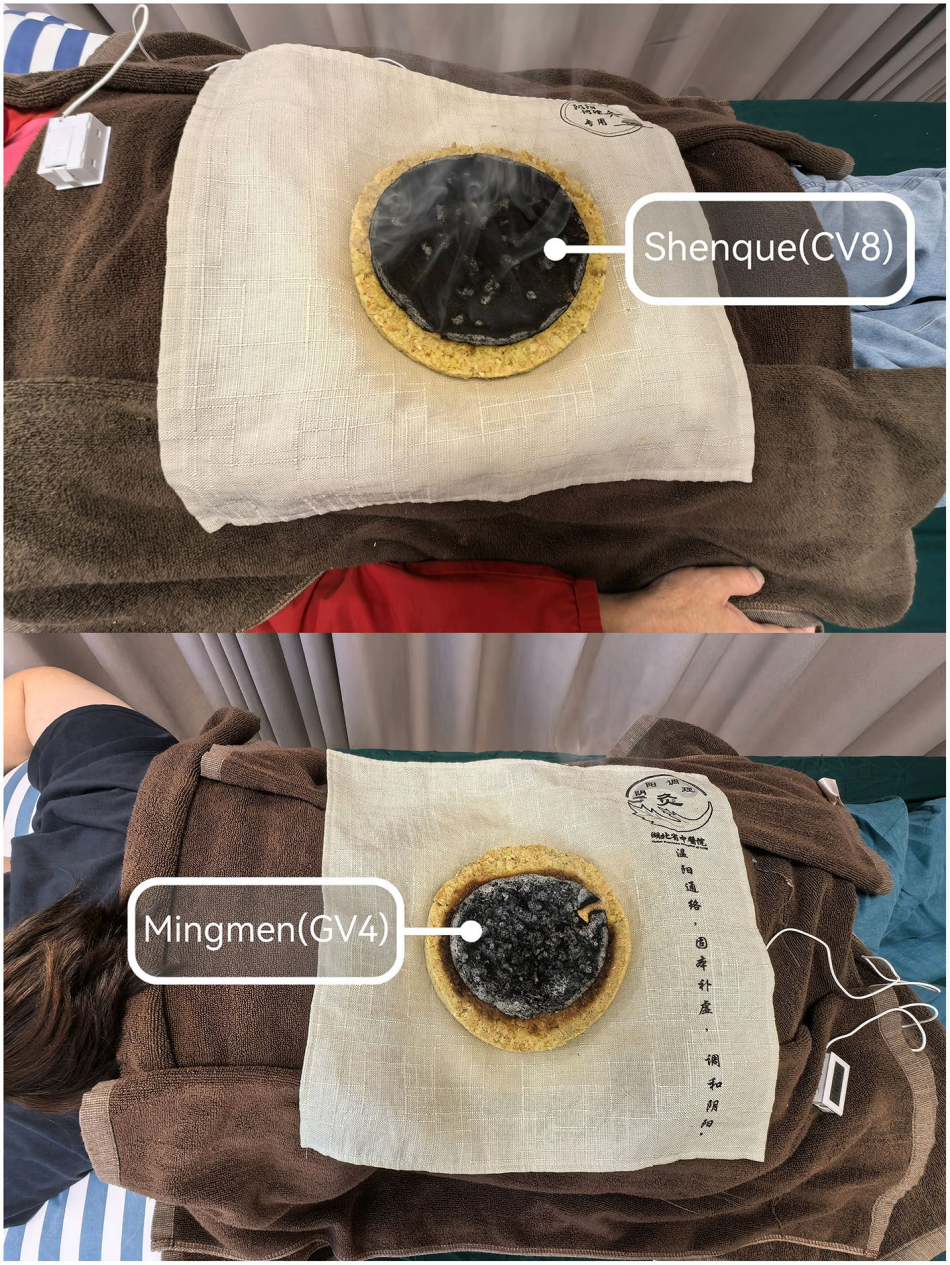
The procedure of Yin-Yang Regulating Moxibustion. A compressed moxa disc is positioned on top of the ginger base at Shenque (CV8) or Mingmen (GV4).

In the prone position, treatment is centered on the Mingmen acupoint (GV4), covering an approximate 8–10 cm radius. In the supine position, moxibustion targets the Shenque acupoint (CV8) (Figure 4). The two positions alternate weekly throughout the intervention (GV4 on Week 1, CV8 on Week 2, and so on). The alternating CV8/GV4 treatment schedule will be implemented according to a predefined protocol. CV8 and GV4 were selected as complementary core acupoints according to the TCM therapeutic principle of warming yang and tonifying the kidney, which corresponds to the kidney yang deficiency pattern commonly described in DOR.

At the beginning of each session, the practitioner molds a ginger paste disc approximately 3 cm thick (around 700 g in weight and 10 cm in diameter) using a circular mold and places it over the selected acupoint. A compressed moxa disc (14.5 cm in diameter, approximately 30 g) is then positioned on top of the ginger base and ignited. Each treatment lasts for 40 min. Throughout the session, the practitioner monitors the local skin temperature using a digital thermometer with a probe, maintaining a stable temperature between 40 °C and 44 °C. If the temperature rises above the target range or causes discomfort, a clean cotton pad is inserted between the ginger and the skin to reduce heat. To reduce smoke-related exposure, the smoke purification and local exhaust device will be operated throughout all moxibustion sessions, with routine room ventilation maintained as needed (30, 31). If a participant reports discomfort related to moxa smoke, the practitioner will promptly adjust the local ventilation conditions. At each session, the practitioner will record the participant’s treatment position, acupoint, treatment duration, temperature range, and any deviations from the planned procedure to ensure consistency and feasibility of the 16-week YYRM intervention. All treatments are performed by licensed Traditional Chinese Medicine practitioners trained specifically in YYRM methodology. The detailed procedure is provided in Supplementary S3 File.

#### 2.6.2 Control group

The control group will receive weekly lifestyle intervention sessions over the same period, covering dietary guidance, lifestyle modification, emotional management, and avoidance of environmental toxins. No moxibustion interventions will be administered to the control group.

### 2.7 Outcome measurement

#### 2.7.1 Primary outcome

AFC: At baseline (week 0), after the 16-week intervention (week 16) and at the end of the follow-up period (week 32), transvaginal ultrasound examinations will be performed to assess the number of 2–9 mm antral follicles in both ovaries. The total AFC will be calculated as an important indicator of ovarian reserve function.

#### 2.7.2 Secondary outcomes

(1) Serum levels of AMH, FSH, LH: At baseline (week 0), after the 16-week intervention (week 16) and at the end of the follow-up period (week 32), venous blood samples will be collected during the early follicular phase (days 2–4 of the menstrual cycle) to measure AMH, FSH, and LH levels (32, 33).

(2) Scale Assessments: The following validated scales will be used to assess outcomes: Modified Kupperman Index (mKMI) (34): To evaluate the severity of menopausal symptoms. Hamilton Anxiety Scale (HAMA) (35) and Hamilton Depression Scale (HAMD) (36): To assess the effects of the intervention on anxiety and depressive symptoms. Menopause-Specific Quality of Life Questionnaire (MENQOL) (37): To assess health-related quality of life during the perimenopausal period. Assessments will be conducted at week 0, week 16, and week 32.

#### 2.7.3 Safety assessment

Adverse events (AEs) will be recorded throughout the intervention and follow-up periods. At each treatment visit, participants will be asked about any AEs they may have experienced. For each reported AE, the following information will be documented: specific symptom(s), onset time, duration, severity, resolution time, and the suspected relationship to the intervention. Particular attention will be paid to potential AEs associated with moxibustion, including those caused by heat or moxa smoke exposure. Commonly reported moxibustion-related adverse effects may include local burns, blisters, skin itching, dizziness, eye swelling, foot pain, dry mouth, constipation, and abdominal pain (38).

#### 2.7.4 Rs-fMRI

Resting-state functional magnetic resonance imaging (rs-fMRI) will be conducted at Hubei Provincial Hospital of Traditional Chinese Medicine. Each participant will undergo two MRI scans, at baseline (week 0) and after the 16-week intervention (week 16). For each participant, longitudinal MRI scans will be scheduled at a similar time of day whenever possible to minimize circadian-related variability in resting-state neural activity. Relevant pre-scan lifestyle factors, including sleep status, caffeine and alcohol intake, smoking status, and vigorous physical activity, will be recorded and considered when interpreting rs-fMRI findings. Before scanning, participants will be instructed to remove all metallic objects and wear noise-reducing earplugs. During scanning, participants will lie in a supine position, remain still, and keep their eyes closed while staying awake and relaxed. Foam cushions will be used to fix the head in place to minimize motion artifacts. This study will collect multimodal neuroimaging data to investigate the potential mechanisms by which the intervention modulates brain function and structure.

All imaging will be performed on a Siemens MAGNETOM Skyra 3.0 T MRI scanner using a 32-channel head coil. The imaging protocol includes a high-resolution T1-weighted structural sequence, rs-fMRI and diffusion weighted images (DWI) sequence. To exclude intracranial structural abnormalities that may interfere with the results, anatomical evaluation will be performed concurrently.

Scanning Parameters: High-resolution T1-weighted structural imaging (T1WI): Acquisition time = 280 s matrix = 256 × 256 × 176 slices, repetition time (TR) = 2000 ms, echo time (TE) = 2.23 ms, flip angle = 8°, field of view (FOV) = 240 × 240 × 240, voxel size = 0.9 × 0.9 × 0.9 mm. fMRI scan: acquisition time = 488 s, matrix = 64 × 64 × 36 slices, TR = 2000 ms, TE = 30 ms, dynamic scans = 240, flip angle = 90°, FOV = 224 mm × 224 mm × 224 mm, voxel size = 3.5 mm × 3.5 mm × 4.0 mm. DWI: acquisition time = 945 s, matrix = 112 × 112 × 46 slices, TR = 6,600 ms, TE = 107 ms, FOV = 224 mm × 224 mm × 224 mm, voxel size = 2.0 mm × 2.0 mm × 3.0 mm. A multi-shell protocol was used with b-values of 0, 1,000, and 2000.

Consistent with prior human resting-state fMRI studies investigating hypothalamus-centered functional connectivity (39), a standardized preprocessing and analysis pipeline will be implemented to mitigate the influence of limited spatial resolution and physiological noise. Preprocessing will include removal of the initial volumes to ensure signal stabilization, slice timing correction, rigid-body head motion correction, and spatial co-registration of functional images to the corresponding high-resolution T1-weighted structural images, followed by normalization to standard space. To further reduce non-neuronal signal contributions, nuisance regression will be performed, including signals derived from white matter, cerebrospinal fluid, and motion parameters. Temporal band-pass filtering will be applied to retain low-frequency fluctuations relevant to resting-state functional connectivity, while attenuating high-frequency physiological noise. Spatial smoothing will be conducted using a modest Gaussian kernel to enhance signal-to-noise ratio while preserving anatomical specificity in subcortical regions.

Hypothalamus-centered seed regions will be defined based on established anatomical references in standard space. In accordance with prior human rs-fMRI studies, seed locations will be placed within the medial and lateral hypothalamic regions using standardized MNI coordinates reported in the literature (e.g., medial hypothalamus approximately at *x* = ±4, *y* = −2, *z* = −12; lateral hypothalamus approximately at *x* = ±8, *y* = −10, *z* = −12), with small spherical regions of interest to minimize contamination from adjacent cerebrospinal fluid and surrounding structures. Seed placement will be further constrained by anatomical masks derived from structural images to ensure consistency across participants.

### 2.8 Sample size estimation

The sample size was calculated based on the primary outcome, namely the change in AFC from baseline to week 16. Data from our previous study indicated that the mean AFC level after YYRM treatment in the intervention group was 4.16 ± 0.86, while the change in the waitlist group was 3.00 ± 1.69. Assuming a minimal clinically important difference of 1.8 between the two groups, with a one-sided significance level of *α* = 0.025 and statistical power of 90% (*β* = 0.10), the required sample size was estimated to be 56 participants (28 per group). Considering a potential dropout rate of 15%, the final sample size was increased to 66, with 33 participants in each group. Sample size estimation was performed using PASS15.

### 2.9 Statistical analysis

All data from enrolled participants will be collected and analyzed. Descriptive statistics will be used to summarize baseline characteristics. The Shapiro–Wilk test will be applied to assess the normality of continuous variables. For normally distributed quantitative data, results will be presented as mean ± standard deviation (SD). For non-normally distributed data, results will be presented as median and interquartile range (IQR). Categorical variables will be expressed as frequencies and percentages, and between-group comparisons will be made using the chi-square test or Fisher’s exact test, as appropriate. To assess the treatment effects over time, linear mixed-effects models will be used to analyze repeated measures, accounting for both fixed effects (group, time, group × time interaction) and random effects. Both intention-to-treat (ITT) and per-protocol (PP) analyses will be conducted. For the ITT analysis, missing data will be handled using the Last Observation Carried Forward (LOCF) method, as previously used in acupuncture studies (40, 41). The PP analysis will include participants who complete the intervention and outcome assessments without major protocol deviations. Statistical significance will be defined as a two-sided *p*-value <0.05. All statistical analyses will be performed using SPSS version 24.0.

Lifestyle adherence scores will be summarized during the 16-week intervention period. The mean weekly adherence score will be calculated for each participant based on the Healthy Lifestyle Adherence Scoring System. Between-group differences in lifestyle adherence will be evaluated descriptively. Where appropriate, the mean lifestyle adherence score will be included as a covariate in adjusted analyses for clinical outcomes and rs-fMRI-related exploratory analyses. Sensitivity analyses may also be conducted to examine whether the main findings remain robust after accounting for lifestyle adherence or excluding participants with marked lifestyle non-compliance.

fMRI data will be processed using MATLAB R2017b in conjunction with the CONN functional connectivity toolbox (version 20). Standard preprocessing procedures will include slice timing correction, realignment, spatial normalization to MNI space, smoothing, artifact detection, linear trend and covarients removal, and temporal band-pass filtering. Hypothalamus and other relevant brain regions will be defined as the primary region of interest (ROI) for functional connectivity analysis.

To explore the association between central functional connectivity changes and peripheral ovarian reserve-related biomarkers, an exploratory correlation analysis will be performed. For participants with available imaging and peripheral biomarker data at both baseline and week 16, changes in hypothalamus-centered functional connectivity values will be calculated and correlated with changes in AFC, FSH, and LH from baseline to week 16. Pearson correlation analysis will be used when the variables are normally distributed, whereas Spearman rank correlation analysis will be applied for non-normally distributed variables. When multiple functional connectivity clusters or multiple peripheral biomarkers are examined, appropriate multiple-comparison correction will be applied to adjust for multiple comparisons. These analyses will be considered exploratory and hypothesis-generating, and will not be interpreted as evidence of causal brain-ovary regulation.

## 3 Discussion

With the societal trend toward delayed marriage and childbirth, female fertility is declining with advancing age. Coupled with unhealthy lifestyle habits and excessive work-related stress, the incidence of DOR has been rising steadily and is now increasingly observed in younger women.

This protocol evaluates the potential effects of YYRM in patients with DOR using a multidimensional assessment framework. AFC, AMH, FSH, and LH provide complementary information on ovarian reserve status from morphological, biochemical, and endocrine perspectives. AFC reflects the recruitable follicle pool, AMH provides relatively stable biochemical information on ovarian reserve, and FSH and LH are more closely related to HPO-axis feedback regulation (42). Therefore, these indicators together may help characterize ovarian reserve changes after YYRM more comprehensively than any single marker alone. The rs-fMRI component of this study is exploratory and is intended to investigate potential central regulatory pathways in patients with DOR, rather than to serve as the primary endpoint or to establish confirmatory evidence of brain-ovary causality.

According to the TCM theory, DOR is fundamentally associated with kidney yang deficiency, which reflects a functional insufficiency in sustaining reproductive vitality rather than a localized ovarian disorder (43). Based on this pathogenesis, YYRM was designed according to the therapeutic principle of warming yang and tonifying the kidney. Therefore, the selection of acupoints in this protocol emphasizes points traditionally associated with supporting kidney yang and reproductive function.

In this study, Shenque (CV8) and Mingmen (GV4) were selected as primary acupoints for intervention. CV8, located in the center of the umbilicus and associated with the Conception Vessel. It is traditionally regarded as a key site for supporting vital essence and reproductive function (44). GV4, an acupoint of the governor vessel, is located at the lumbar spine. Stimulating GV4 can tonify and nourish the Kidney Yang. Stimulation at these sites may engage somatosensory and autonomic afferent pathways that influence hypothalamus-centered neural activity (45, 46). Through this pathway, moxibustion may modulate central neuroendocrine regulation related to ovarian function.

The hypothalamus was selected as the primary seed region because it is a key central node in reproductive neuroendocrine regulation and plays a direct role in gonadotropin-releasing hormone signaling within the HPO axis (47). For a hypothesis-driven seed-based rs-fMRI design, the hypothalamus provides a biologically plausible entry point for exploring the potential central mechanisms linking moxibustion and ovarian function. This choice is also consistent with a DOR neuroimaging study protocol that adopted a hypothalamus-centered framework (15).

The primary objective of this study was to assess the clinical efficacy of moxibustion in women with DOR and to investigate its potential central mechanisms using fMRI. By incorporating fMRI as a complementary neuroimaging technique, we aimed to explore the central regulatory pathways associated with ovarian function and provide novel insights into the neuroendocrine basis of DOR rather than to establish causal mechanisms. Our findings are expected to enhance understanding of the therapeutic potential of moxibustion in improving ovarian reserve and to identify objective, neuroimaging-based markers for evaluating treatment response. Such evidence may further support the standardization of moxibustion assessment protocols in clinical trials and promote its broader clinical application in reproductive medicine.

This study innovatively integrates fMRI to investigate the impact of moxibustion on brain activity patterns. However, there are still several limitations in this trial: First, this study adopts an open-label design and does not include a sham moxibustion or placebo control. Due to the thermal and sensory characteristics of moxibustion, double-blinding of both participants and practitioners was not feasible, which may introduce expectancy bias. Therefore, the present study cannot fully separate the specific effects of YYRM from nonspecific effects associated with receiving active treatment. Second, our study is a single-center trial conducted solely in one hospital in China. thus, the findings require validation in larger, more diverse populations. Third, the sample size was relatively small, and this was a single-center, open-label study. These factors may limit the strength of the evidence generated by the present study. Therefore, the findings of this trial should be regarded as preliminary and interpreted with caution. Fourth, while the hypothalamus serves as a focused entry point for exploring ovarian-related central regulation, studies have also shown that other regions and networks may play a role in the central neuroendocrine regulation (48–50). We will consider investigating these regions in future studies, if necessary, to further elucidate the underlying mechanisms.
