# Supplementary material for: Clinical efficacy and mechanism exploration of Moxibustion for diminished ovarian reserve: a randomized controlled trial protocol
Source: Front Public Health. 2026 Jul 16;14:1805995. doi: 10.3389/fpubh.2026.1805995 (PMC13422477; doi:10.3389/fpubh.2026.1805995)
Supplement: Supplementary file 2 [file Table_1.DOCX]

**Healthy Lifestyle Adherence Scoring System (16-week Intervention)**

Participants will receive lifestyle education focusing on four domains: dietary modification, physical activity, sleep–wake regulation, and avoidance of harmful substances. Adherence will be evaluated weekly during the 16-week intervention period. Each domain will be scored on a 0–2 scale, with higher scores indicating better adherence. The weekly total score ranges from 0 to 8 and we conduct adherence assessments weekly.

| **Domain** | **Recommendation** | **Scoring method** | | **Weekly Range** |
| --- | --- | --- | --- | --- |
| Dietary modification | 1. Increase vegetable protein, whole grains, soy products, monounsaturated fats; 2. reduce trans fats and high-glycemic load foods | ≤2 days/week adherence | 0 | 0-2 |
|  |  | 3–4 days/week adherence | 1 |  |
|  |  | ≥5 days/week adherence | 2 |  |
| Physical activity | 1. Moderate-intensity aerobic exercise≥150 min/week over ≥5 days. 2. Intensity classified as light (<3 METs), moderate (3–6 METs), or vigorous (>6 METs) | <75 min/week or <3 days, regardless of intensity. | 0 | 0-2 |
|  |  | 75–149 min/week moderate, or <75 min vigorous, 3–4 days | 1 |  |
|  |  | ≥150 min/week moderate, or ≥75 min vigorous, ≥5 days | 2 |  |
| Sleep–wake schedule | 1. Sleep 7–8 h/night. 2. Bedtime before 23:30. 3. Limited screen exposure, ≤1 night shift/week. | Meets ≤1 criterion | 0 | 0-2 |
|  |  | Meets 2 criteria | 1 |  |
|  |  | Meets≥3 criteria | 2 |  |
| Avoidance of harmful substances | Avoid smoking; alcohol ≤1 occasion/week | Current smoker or alcohol ≥2 times/week | 0 | 0-2 |
|  |  | Non-smoker + occasional alcohol ≤1/week | 1 |  |
|  |  | Non-smoker + no alcohol | 2 |  |
